# Exploring the biosynthetic potential of Korean Actinobacteria for antibacterial metabolite discovery

**DOI:** 10.71150/jm.2504002

**Published:** 2025-09-30

**Authors:** Sehong Park, Hyun-Woo Je, Yujin Cha, Boncheol Gu, Yeojeong Cho, Jin-Il Kim, Ji Won Seo, Seung Bum Kim, Jino Son, Moonsuk Hur, Changmin Sung, Min-Kyu Oh, Hahk-Soo Kang

**Affiliations:** 1 Department of Biomedical Science and Engineering, Konkuk University, Seoul 05029, Republic of Korea; 2 Department of Chemical and Biological Engineering, Korea University, Seoul 02841, Republic of Korea; 3 Doping Control Center, Korea Institute of Science and Technology, Seoul 02792, Republic of Korea; 4 Department of Microbiology and Molecular Biology, Chungnam National University, Daejeon 34134, Republic of Korea; 5 Biological Resources Assessment Division, National Institute of Biological Resources, Incheon 22689, Republic of Korea

**Keywords:** actinobacteria, biosynthetic gene clusters, antibacterial activity, svetamycins

## Abstract

Actinobacteria, a phylum of Gram-positive bacteria, are renowned for their remarkable ability to produce antibacterial natural products. The National Institute of Biological Resources (NIBR) of Korea maintains a collection of Korean native actinobacteria. In this study, we explored the phylogenetic and biosynthetic diversity of the NIBR actinobacteria collection to assess its potential as a source of new antibacterial natural products. A 16S rDNA-based phylogenetic analysis revealed a high level of genetic diversity within the collection, with a predominance of *Streptomyces*, along with rare actinobacterial genera such as *Kitasatospora* and *Micromonospora*. Additionally, genetic network analysis of biosynthetic gene clusters (BGCs) from 15 sequenced NIBR actinobacterial strains demonstrated extensive BGC diversity, with many clusters identified as cryptic. Screening of culture extracts for antibacterial activity, followed by dereplication of active extracts, suggested the presence of potentially novel antibacterial natural products. Activity-guided isolation and whole-genome sequencing of the active strain KU57 led to the isolation of one new and three known svetamycin congeners along with their BGC. Overall, our findings highlight the NIBR actinobacteria collection as a valuable source for the discovery of new antibacterial natural products.

## Introduction

The global rise of antibiotic-resistant bacteria poses a significant challenge to public health (Darby et al., 2023). The World Health Organization (WHO) has identified antimicrobial resistance as one of the most critical threats to the global public health and development (Ahmed et al., 2024). To address this crisis, the continuous development and introduction of new antibiotics are essential to combat pathogenic bacteria that have become resistant to all existing antibiotics. Actinobacteria, a phylum of Gram-positive bacteria, are renowned for their capacity to produce antibacterial metabolites (Bérdy, 2012; Parra et al., 2023) as approximately 75% of antibiotics used in clinics today originated from actinobacteria, especially from the genus *Streptomyces* (Genilloud, 2017). While many antibacterial metabolites have already been discovered, genomic studies reveal that these bacteria still harbor a substantial number of cryptic biosynthetic gene clusters (BGCs) with their products yet to be discovered (Chung et al., 2021). Therefore, continuing exploration of these cryptic BGCs in actinobacteria is vital to ensure a sustainable pipeline of new antibiotics capable of combating the rising threat of antibiotic-resistant pathogens.

Korea is a rich habitat for diverse actinobacteria (Bae et al., 2016), which have proven to be a prolific source of biologically active natural products (Jang et al., 2018; Kim et al., 2015; Son et al., 2017, 2018). Therefore, establishing a dedicated Korean actinobacteria culture collection, coupled with systematic genome sequencing, would facilitate the discovery of new antibiotics from cryptic BGCs hidden in the genomes of Korean actinobacterial genomes. The National Institute of Biological Resources (NIBR) of Korea, a government organization under the Ministry of Environment, manages diverse collections of Korea’s native biological resources and conducts research on their biodiversity (Hur et al., 2016). Among these is a collection of Korean native actinobacteria, which remains largely unexplored as a source of biologically active natural products. As the NIBR actinobacteria collection continues to grow, they have recently initiated a project to investigate their metabolic diversity and industrial potential. As part of this effort, we obtained the actinobacterial collection from NIBR and conducted their genetic and biosynthetic analyses, focusing on evaluating their potential to produce antibacterial natural products.

A 16S rDNA sequence-based phylogenetic analysis revealed the high genetic diversity of the NIBR actinobacterial collection, which is predominantly composed of *Streptomyces* species, along with smaller proportions of rare genera such as *Kitasatospora*, *Nocardia*, and *Micromonospora*. Analysis of BGC abundance and genotype diversity across 12 sequenced genomes demonstrated the collection’s high biosynthetic potential, with many BGCs remaining cryptic. Preliminary antibacterial screening of organic culture extracts showed a high hit rate, with over half of the strains exhibiting activity. Dereplication of active extracts, followed by activity-guided isolation, led to the identification of four svetamycin congeners, including one new and three known compounds. Antibacterial assays of the isolated compounds provided insights into their structure-activity relationships. This study highlights the value of NIBR’s actinobacteria collection as a promising source of new antibacterial natural products.

## Materials and Methods

### Culture condition and gDNA isolation

Korean native actinobacterial strains were obtained from the NIBR culture collection and routinely maintained on ISP4 agar media. For genomic DNA (gDNA) isolation, the strains were cultured in the TSB (Tryptic Soy Broth) liquid media for 3 days, and high-quality gDNAs were extracted for whole genome sequencing using the salting-out method previously described (Pospiech and Neumann, 1995). For metabolite production, the strains were grown in 50 ml of R5A liquid media at 30°C with shaking (200 rpm). After 7–10 days, the cultures were extracted with an equal volume of ethyl acetate at neutral and acidic pH. The extracts were dried in vacuo and then dissolved in a small volume of either dimethyl sulfoxide (DMSO) or methanol (MeOH) for activity or HPLC analyses.

### 16S rDNA phylogeny

The 16S rDNAs of the NIBR strains were PCR-amplified from gDNAs using the universal primers 27F (5’-AGAGTTTGATCCTGGCTCAG-3’) and 1492R (5’-GGTTACCTTGTTACGACTT-3’) and sequenced by Sanger sequencing (Macrogen). The 16S rDNA gene sequences of the *Streptomyces* type strains were retrieved from the NCBI (National Center for Biotechnology Information) database using the accession numbers reported by Labeda et al. (2012). Sequence alignment was performed using CLC Main Workbench (Qiagen) with default settings. The resulting sequence alignment was used for the construction of the phylogenetic tree in MEGA 11 (Tamura et al., 2021). The evolutional relationships were inferred using the neighbor-joining method with the Jukes-Cantor distance measure and 1,000 bootstrap replications. The *E. coli* 16S rDNA sequence was used as the outgroup. The tree was visualized using the Interactive Tree of Life (iTOL version 6) (Letunic and Bork, 2024). The *Kitasatospora* subtree was constructed in the same way as described above.

### Whole genome sequencing

Sequencing of the four genomes was performed on both PacBio and Illumina platforms (Macrogen). For PacBio sequencing, sequencing templates were prepared using the SMRTbell Template Prep Kit according to the guide and sequenced on the PacBio RS system. In the case of Illumina sequencing, sequencing libraries were prepared by random fragmentation of genomic DNAs, followed by end-repair and ligation with the sequencing adapter on both ends. The prepared libraries were loaded onto the flow cells where the libraries were captured on surface-bound oligos complementary to the library adapters. Library fragments were amplified into clonal clusters via bridge amplification and sequenced using Illumina SBS (sequencing by synthesis) technology. De novo assembly of genomic DNA sequences was performed using both long reads generated by PacBio sequencing and short reads generated by Illumina sequencing. First, pre-assembly was carried out by mapping single-pass long reads to seed reads (the longest portion in the read length distribution), and then the assembly was performed using HGAP3 (Hierarchical Genome Assembly Process 3) (Chin et al., 2013). After the assembly, the Illumina short reads were aligned to the PacBio draft assembly for error correction using the Pilon error correction tool (Walker et al., 2014). The de novo assembly process generated one to six high-quality contigs.

### BGC diversity analysis

BGC abundance represents the number of BGCs per genome (Chung et al., 2021). The annotated genome sequences were used for BGC abundance analysis using antiSMASH (Antibiotics and secondary metabolite analysis shell) version 7.0 (Blin et al., 2023). The “relaxed” detection strictness was applied to all analyses. The BGC types assigned by antiSMASH were manually inspected and re-classified according to our own classification criteria. The resulting dataset comprised of 417 putative BGCs across 15 genome sequences as well as 1,923 previously characterized BGCs from the MIBiG (Minimum Information about a Biosynthetic Gene Cluster) database version 3.1 (Terlouw et al., 2022) was subjected to the genetic similarity network analysis using the BiG-SCAPE (biosynthetic genes similarity clustering and prospecting engine) pipeline (Navarro-Muñoz et al., 2020). The resulting sequence similarity network with a default similarity score cutoff (c = 0.3; threshold of 30%) consisted of 609 unique nodes and 1,655 edges. The obtained sequence similarity matrices were then visualized using Cytoscape 3.10.3 (Shannon et al., 2003).

### Antibacterial activity screening

The extracts of 50 ml cultures were dissolved in 100 μl of DMSO and used for antibacterial activity screening. The disk diffusion method (Desbois and Smith, 2015) was employed to evaluate antibacterial activity against *Bacillus* subtilis, *Klebsiella pneumoniae*, *Staphylococcus aureus*, and Methicillin-resistant *S. aureus*. These strains were cultured overnight at 37°C in LB media with shaking (200 rpm). One milliliter of the overnight cultures was inoculated into 250 ml autoclaved LB agar media, which was cooled to 50°C, to prepare the infused media. Then, 6 mm filter paper disks were placed on top of the bacteria-infused agar, and 10 μl of the extracts were applied to the filter papers. The agar plates were incubated overnight at 30°C, and the diameter of the inhibition zones was measured to quantitatively assess the antibacterial activity of each extract.

### Dereplication

Dereplication of extracts with potent antibacterial activity was performed using both LC-MS/MS and NMR analyses. For LC-MS/MS-based dereplication, low-resolution LC-MS and high-resolution LC-MS/MS analyses were performed using InfinityLab LC/MSD (Agilent) and Vanquish-Q Exactive (Thermo) mass spectrometry, respectively. MS/MS data was subjected to GNPS (Global Natural Products Social molecular networking) analysis (Nothias et al., 2020). For NMR-based dereplication, 1D and 2D NMR spectra including ^1^H NMR, ^13^C NMR, COSY, HSQC, HMBC, and NOESY spectra were obtained on a Bruker Avance III 600 MHz NMR spectrometer. ^1^H and ^13^C NMR chemical shifts were referenced to the DMSO-*d*_6_ solvent signals (*δ*_H_ 2.50 and *δ*_C_ 39.5, respectively). The HMBC spectrum was recorded with an average ^3^*J*_CH_ of 8 Hz and the HSQC spectrum was measured with an average ^1^*J*_CH_ of 145 Hz.

### Compound isolation

Svetamycins were isolated from 500 ml cultures (50 ml × 10, 200 rpm at 30°C) of *Streptomyces* sp. KU57 (NIBRBAC000002345). The cultures were harvested after 8 days, extracted with the same volume of ethyl acetate and dried in vacuo. The resulting extract was fractionated using Sep-Pak C18 cartridges (Waters) eluting with 0%, 20%, 40%, 60%, 80%, and 100% acetonitrile in water. The active fractions were combined and subjected to preparative HPLC. Svetamycins were isolated using reversed-phase HPLC (C18 column, 10 mm × 250 mm, 3.5 ml/min) and 40% aqueous acetonitrile with 0.1% trifluoro acetic acid.

### Antibacterial assay

Minimal inhibitory concentrations (MIC) for svetamycin A–C (1–3) and deschlorosvetamycin C (4) against *Escherichia coli*, *K. pneumoniae*, *Salmonella typhimurium*, *B. subtilis*, *Kocuria rhizophila*, *S. epidermidis*, *S. aureus*, and Methicillin-resistant *S. aureus* (MRSA). Each bacterial strain was grown overnight in appropriate media. Overnight cultures were diluted 1000-fold and transferred to 96-well microtiter plates (75 μl/well). Compounds were dissolved in DMSO, added to the media in the first well of the row to give a concentration of 50 μg/ml and serially diluted 2-fold/well across the plate. These solutions (75 μl) were then added to the corresponding well in an assay plate and incubated at 30°C overnight without shaking. Bacterial growth was visually inspected and the last well with no bacterial growth was reported as the MIC.

## Results and Discussion

### Phylogenetic diversity of NIBR’s actinobacteria collection

**Fig. 1.**
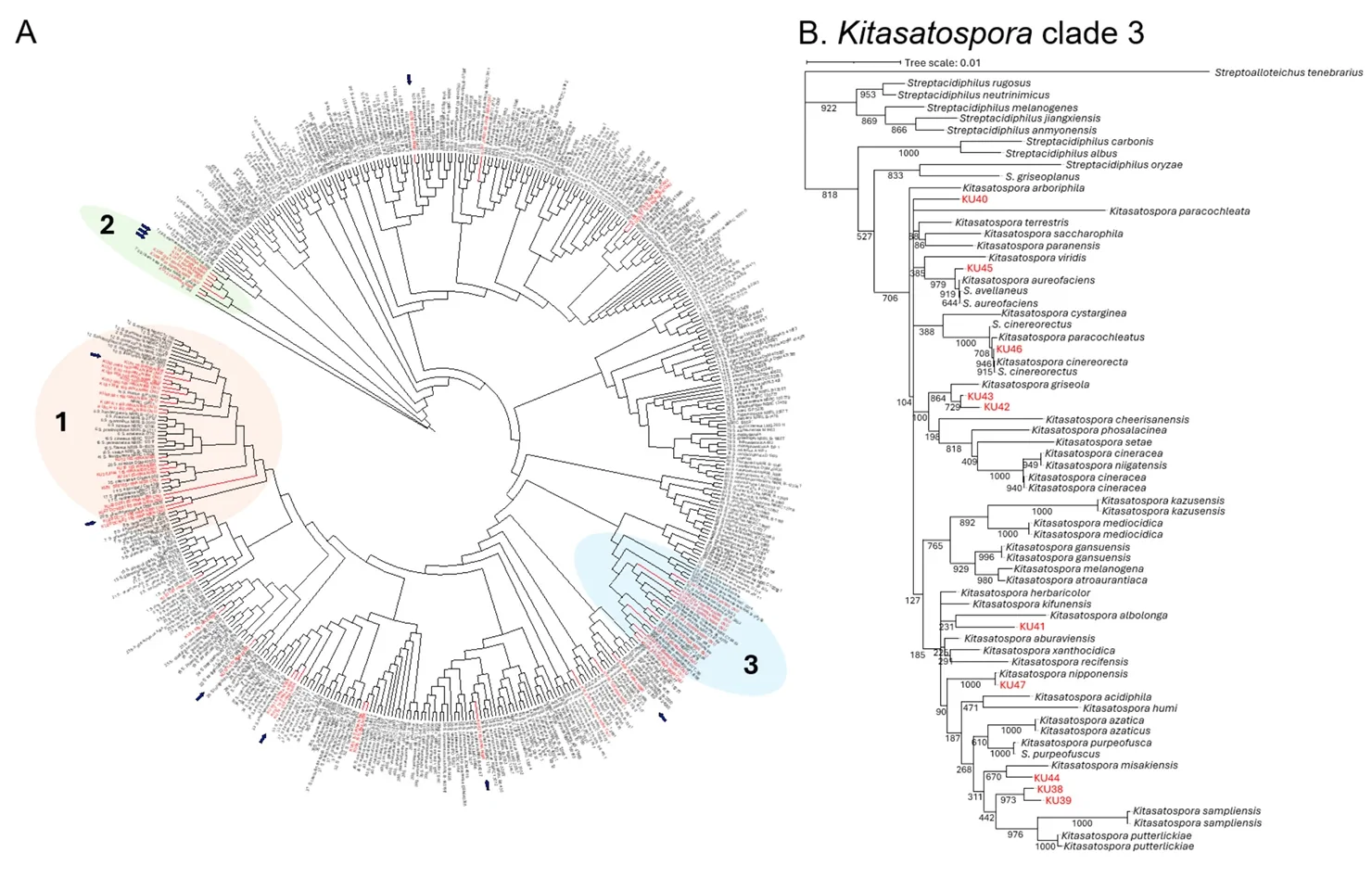
16S rDNA-based biodiversity analysis of NIBR actinobacterial strain collection. (A) Neighbor-joining phylogenetic tree constructed using 16S rDNA sequences of actinobacterial strains. Bootstrap values were calculated from 1,000 bootstrap replications to assess the reliability of the tree topology. NIBR actinobacterial strains were highlighted in red, and strains with genome sequences available are marked with arrows. (B) Clade 1 sub-tree. Clade 1 is comprised of strains from the rare actinobacterial genus *Kitasatospora*.

To gain insight into phylogenetic diversity of the NIBR’s Korean actinobacteria collection, we conducted 16S rDNA sequence analysis on 60 actinobacterial strains (Table S1). Nearly full-length 16S rDNA sequences (~1.5 kb) were amplified from genomic DNAs using universal primers 27F and 1492R. We then constructed a comprehensive phylogenetic tree, incorporating the NIBR’s actinobacterial strains and reference actinobacterial type strains retrieved from GenBank (Fig. 1A) (Labeda et al., 2012). The neighbor-joining tree was built from the alignment of 16S rDNA sequences using the Jukes-Cantor model with 1,000 bootstrap replications (Tamura et al., 2021). Rare actinobacterial species belonging to the genera *Micromonospora* and *Nocardioides* were excluded from the tree due to their distant evolutional relationship with the most representing actinobacterial genus *Streptomyces*. The resulting phylogenetic tree revealed high genetic diversity within the NIBR’s actinobacteria collection, with strains widely distributed across the tree. Notably, several clades, designated clades 1, 2, and 3, were enriched with NIBR strains (Fig. 1A).

The clade 1 contains the largest number of NIBR strains, with 19 strains, all classified as the genus *Streptomyces*. On an evolutionary time scale, this clade represents the most recent diversification from a common ancestor. Several NIBR strains formed distinct subclades from the known *Streptomyces* type strains including one comprising KU32, KU49, KU22, and KU55 and another containing KU30 and KU35, which might represent Korean indigenous *Streptomyces* species. The clade 2 is the most outgroup in the phylogenetic tree and contains a total of 6 NIBR strains. The only known type strain in this clade is *Streptomyces libani*, which was also isolated from the soil collected at Young-Jong Island in Korea (Kim and Hwang, 1997). This strain was previously characterized to produce the antibiotic oligomycin A (Kim et al., 1999). These results suggest that the clades 1 and 2 together might represent Korean indigenous *Streptomyces* lineages. The clade 3 contains 10 NIBR strains cladding with the known *Kitasatospora* type strains (Fig. 1B). *Kitasatospora* is a rare actinobacterial genus distinct from *Streptomyces*, with only 40 species identified to date, according to the NCBI taxonomy database (Takahashi, 2017). The NIBR strains that fell within the *Kitasatospora* clade showed a distant relationship with the known *Kitasatospora* species except for the strains KU46 and KU47, which tightly cladded with *Kitasatospora cinereorecta* and *Kitasatospora nipponensis*. As *Kitasatospora* have been a source of many biologically active natural products, the NIBR strains in this clade would potentially produce new natural products. Overall, the phylogenetic analysis demonstrates that the NIBR actinobacteria collection represents the phylogenetically diverse actinobacterial strains, with some clades potentially representing the Korean indigenous lineages.

### Biosynthetic diversity of NIBR’s actinobacteria collection

**Fig. 2.**
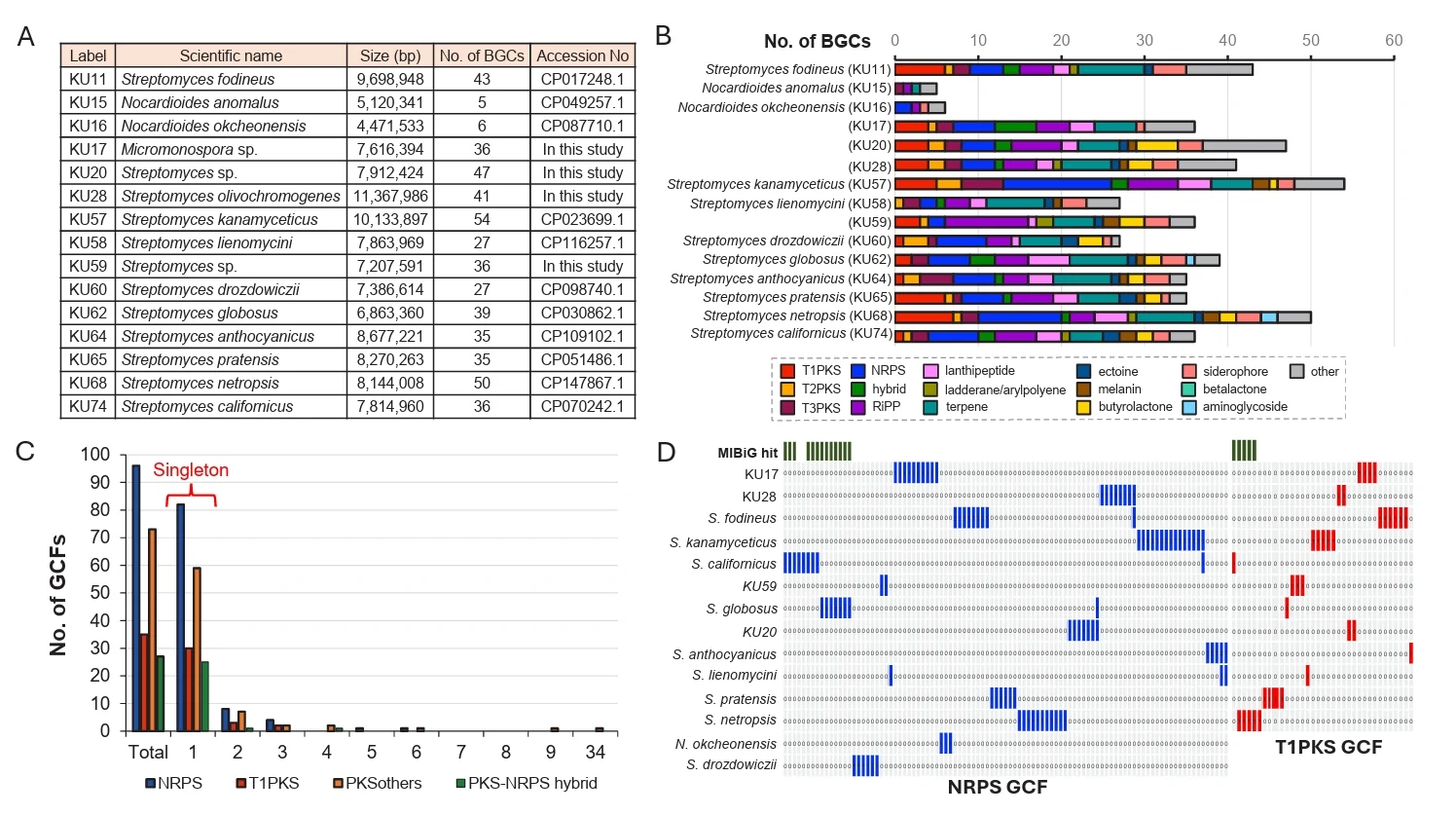
BGC diversity of NIBR actinobacterial collection using sequenced genomes. (A) The list of sequenced NIBR actinobacterial genomes. Of the 15 genome sequences used for BGC diversity analysis, 11 genome sequences were retrieved from the GenBank database, and 4 genomes were sequenced in this study. *S. kanamyceticus* possesses the largest genome and the largest number of BGCs. (B) Comparison of BGC distribution across different BGC families. BGCs identified from antiSMASH analysis were re-classified according to our classification criteria. (C) Prevalence analysis of GCFs across 11 genomes. The singletons are unique GCFs only present in a single genome, whereas shared GCFs are present in multiple genomes. (D) GCF absence/presence analysis. The presence of GCF was highlighted red for T1PKS GCFs and blue for NRPS GCFs. GCF clustered with known BGCs from MIBiG database was highlighted green on the top. Only few GCFs were clustered with known BGCs.

The biosynthetic potential of microbial species can be assessed by analyzing BGC abundance and their genotype diversity. Therefore, we first examined the BGC abundance of NIBR’s actinobacterial strains using sequenced genomes. A total of 11 genome sequences were available in the GenBank database, and we sequenced the genomes of four additional NIBR strains (KU17, KU20, KU28, and KU59) (Fig. 2A). These 15 high-quality genome sequences were subjected to BGC abundance analysis using antiSMASH (Version 7.0) with a detection strictness set to “relaxed” (Fig. 2B; Blin et al., 2023). A BGC abundance is defined as the number of BGCs identified in a single genome, representing the biosynthetic potential of microbial species (Chung et al., 2021). The analysis revealed that the species belonging to the genus *Nocardioides* exhibited the lowest BGC abundance, with only five BGCs identified per genome on average. The genome of KU17 is the only genome belonging to the genus *Micromonospora* and showed a higher BGC abundance (36 BGCs) compared to that of *Nocardioides*. As expected, *Streptomyces* genomes showed a wide range of BGC abundance, with the number of BGCs per genome varying from 27 to 54. The genome of *S. kanamyceticus* contained the largest number of BGCs (54 BGCs). Interestingly, the difference in BGC abundance correlated with variations in the number of PKS (polyketide synthase) and NRPS (non-ribosomal peptide synthetase) BGCs in our dataset (Fig. 2B). The genome with the highest BGC abundance (KU57) also contained the largest number of NRPS (13 BGCs) and PKS (13 BGCs) BGCs. On the other hand, the number of other BGC families, such as terpene and RiPP (ribosomally synthesized and post-translationally modified peptides) BGCs, were relatively constant regardless of BGC abundance. The BGC abundance analysis highlights the promising biosynthetic potential of the NIBR’s actinobacteria, except for species from the rare genus *Nocardioides*.

**Fig. 3.**
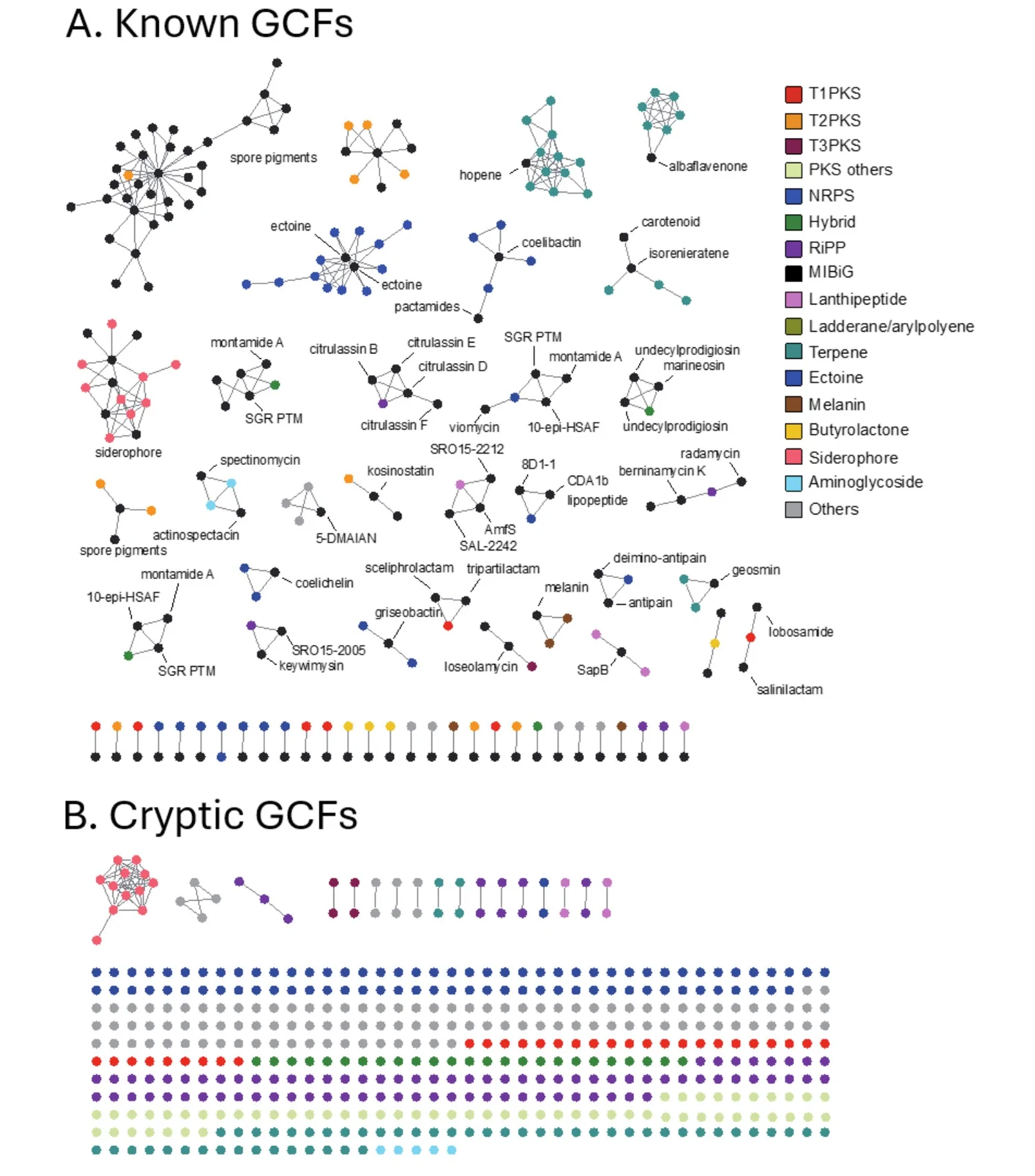
Genetic network analysis of BGCs. Nodes represent individual BGCs, with edge lengths indicating genetic relatedness based on the Jaccard index values calculated using BiG-SCAPE. The genetic network map was visualized using Cytoscape (version 3.8.2). Each network corresponds to a distinct GCF. GCFs were classified as known GCFs if clustered with known BGCs (A) GCFs classified as known are those that cluster with previously characterized BGCs from the MIBiG database. (B) GCFs classified as cryptic do not cluster with any known BGCs, indicating their potential to produce novel natural products.

To evaluate the diversity of BGC genotype, we next conducted a genetic similarity network analysis of BGCs identified in the NIBR’s actinobacterial genomes using the BiG-SCAPE algorithm (Navarro-Muñoz et al., 2020). BiG-SCAPE clusters BGCs into GCFs (gene cluster families) based on three key metrics: the Jaccard index, adjacency index, and domain sequence similarity. To enable genotype-based dereplication of GCFs, we incorporated 1,923 previously known BGCs from the MIBiG (minimum information about a biosynthetic gene cluster) database into the analysis (Terlouw et al., 2022). The resulting GCFs were categorized as either unique or shared GCFs, depending on their presence in single genome or multiple genomes. Additionally, GCFs were classified as known (Fig. 3A) if clustered with at least one known BGC from the MIBiG database or as cryptic (Fig. 3B) if not clustered with any known BGCs (Fig. 2D). Overall, the BGC clustering resulted in a substantial number of unique GCFs, consisting of single BGCs (singletons in Fig. 2C). This observation aligns with the high phylogenetic diversity of the NIBR actinobacteria collection.

In this analysis, shared GCFs were those encoding common natural products such as spore pigments (T2PKS), hopene (terpene), siderophore and ectoine that are known to be prevalent across the actinobacteria phylum (Donia et al., 2014). Notably, all T1PKS GCFs were unique, and only 5 out of the 31 identified T1PKS GCFs were associated with known BGCs, suggesting that these cryptic T1PKS GCFs potentially encode novel natural products. The most prevalent BGC type in the collection was NRPS with 73 GCFs, of which only five GCFs were linked to known BGCs of coelichelin and coelibactin (Bentley et al., 2002), montamide A (Jiao et al., 2020), griseobactin (Patzer and Braun, 2010), and lipopeptide CDA1b (Hojati et al., 2002). The remaining NRPS GCFs were cryptic, indicating their potential to encode novel natural products. Of the 69 RiPP GCFs identified, the majority were cryptic with only three GCFs associated with known BGCs, encoding keywimysin and citrulassins (Tietz et al., 2017), and berninamycins (Malcolmson et al., 2013). The BGC clustering result suggested that the NIBR’s actinobacteria collection harbors a substantial number of genotypically diverse cryptic GCFs that together represent a potential source of novel natural products.

### Preliminary screening for antibacterial activity

**Fig. 4.**
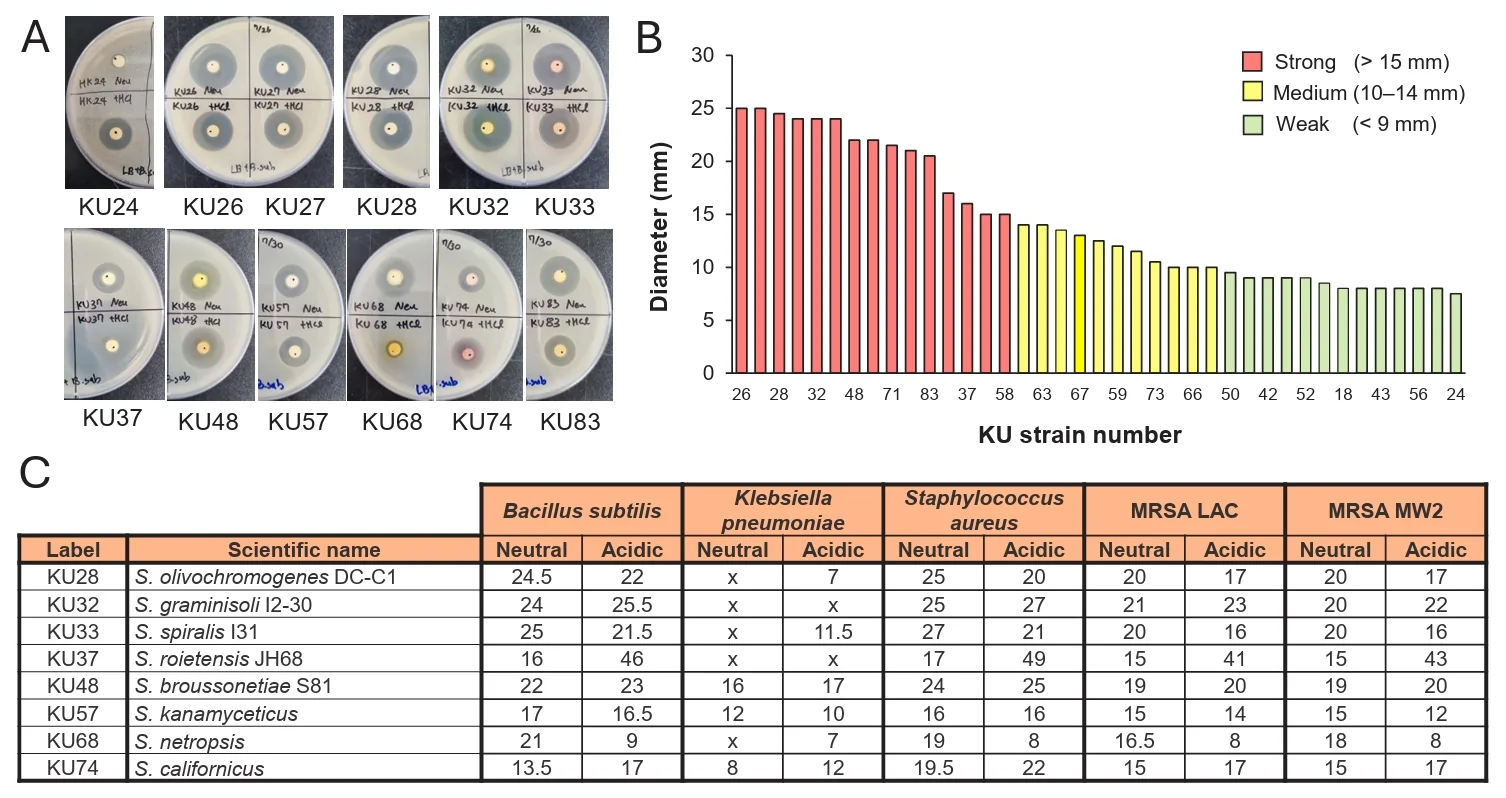
Antibacterial activity of NIBR actinobacterial strains. (A) Disk diffusion assay. Antibacterial activity was evaluated using a disk diffusion assay with ethyl acetate extracts of culture broths. The upper disks on the agar plates displays the activity of neutral extracts, while the bottom disks display the activity of acidic extracts. (B) Antibacterial potency against *Bacillus subtilis*. Extracts were initially screened against *B. subtilis*, and categprozed based on inhibition zone diameters as strong (> 15 mm), medium (10–14 mm), or weak (< 9 mm). (C) Antibacterial spectrum of potent extracts. Extracts classified as possessing strong activity were further tested against *Kocuria rhizophila*, *Staphylococcus aureus*, Methicillin-resistant *S. aureus* (MRSA).

The identification of a substantial number of cryptic BGCs suggested the possibility that some of these BGCs might be expressed under laboratory culture conditions. To explore this possibility, 60 actinobacterial strains obtained from NIBR were cultured in R5A rich medium. The cultures were subsequently extracted with ethyl acetate under both neutral and acidic pH conditions. The resulting extracts were used for antibacterial activity assay (Fig. 4). The assay was performed using the disc diffusion method (Desbois and Smith, 2015), and the antibacterial activity was preliminary tested against *B. subtilis* (Fig. 4A and 4B). Of the 60 strains tested, 39 demonstrated antibacterial activity, demonstrating a high hit rate with more than half of the strains displaying the activity. The antibacterial potency was categorized by measuring an inhibition zone size: strong activity (> 15 mm, red), medium activity (10–15 mm, yellow), and weak activity (< 10 mm, green) (Fig. 4B). Of these, 15 strains exhibited strong antibacterial activity against *B. subtilis*, and thus were further evaluated against diverse pathogens including *K. pneumoniae* ATCC 4352, *S. aureus*, and methicillin-resistant *S. aureus* (MRSA) LAC and MW2 (Fig. 4C). Most of the extracts that were active against *B. subtilis* also displayed antibacterial activity against *S. aureus* and MRSA, though only a few were effective against *K. pneumoniae*. The extracts from the strains KU32 and KU33 exhibited the strongest activity and showed nearly identical chromatograms in HPLC analysis, suggesting that they are genetically identical, and thus are likely to produce the same antibacterial compounds. These preliminary screening results highlight the NIBR’s actinobacterial collection as a promising source of antibacterial natural products.

### Dereplication of extracts with strong antibacterial activity

**Fig. 5.**
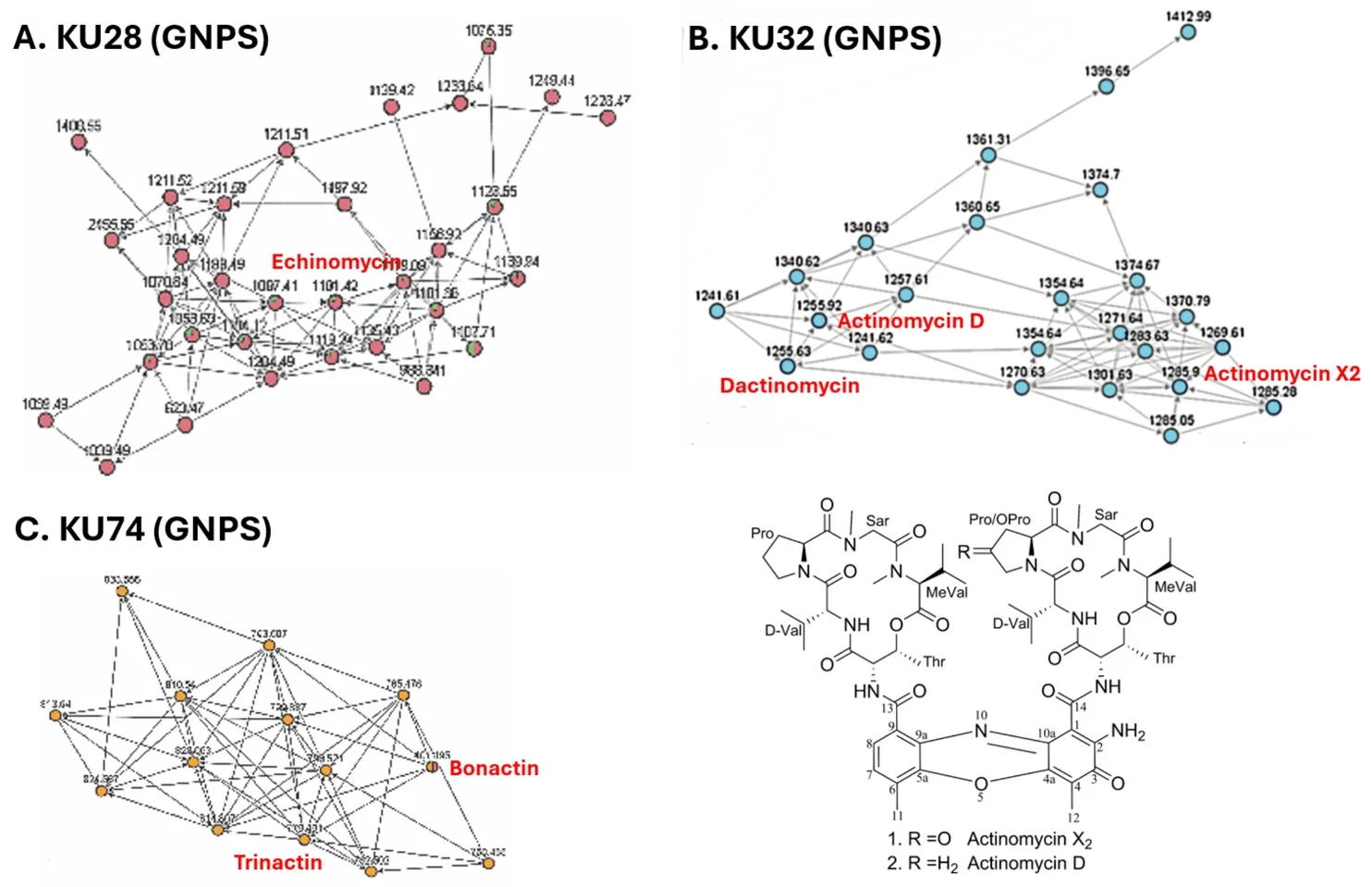
Dereplication of extracts with strong antibacterial activities. (A) Dereplication of the KU28 extract. LC-MS/MS analysis combined with GNPS-based molecular networking revealed that the active compounds in the KU28 extract are likely to be analogs of the known natural product echinomycin. (B) Dereplication of the KU32 extract. Dereplication using the same method indicated that the active compounds in the KU32 extract are likely to be actinomycins D and X2. (C) Dereplication of the KU74 extract. GNPS molecular networking suggested that the active compounds in the KU74 extract are likely to share substructures with bonactin and trinactin.

To see whether the extracts with strong antibacterial activity contain potentially new compounds, we conducted dereplication using LC-MS/MS coupled with GNPS analysis (Fig. 5) (Nothias et al., 2020). In the LC-MS/MS analysis of the KU28 extract, ions with m/z 1119.44, 1141.41, and 1101.43 were identified. GNPS analysis of their MS^2^ fragments revealed clustering with those of the known antibiotic echinomycin (MW 1101.26) (Fig. 5A), indicating active compounds in the KU28 extract to be echinomycin and its derivatives. Similarly, the KU32 extract showed ions with m/z 1271.64, 1269.61, and 1255.63, which corresponded to those of actinomycin X2 (1269.4) and dactinomycin (1255.42). These ions were also clustered together in the GNPS analysis of their MS^2^ fragments (Fig. 5B). In case of the KU74 extract, ions with m/z 782.50, 796.52, 810.53 and 824.55 were detected. However, their mass didn’t match those of any known natural products. Interestingly, the MS^2^ fragments of these ions clustered with those of known ionophores, bonactin and trinactin (Fig. 5C), suggesting that KU74 might produce macrotetrolide antibiotics sharing substructures with these compounds. The remaining KU37, KU48, and KU68 exhibited ion signals in the LC-MS/MS analysis, but their MS^2^ fragments did not cluster with those of any known natural products in the GNPS analysis. This suggests that these strains may produce potentially novel antibiotics.

### Isolation of antibacterial svetamycins

**Fig. 6.**
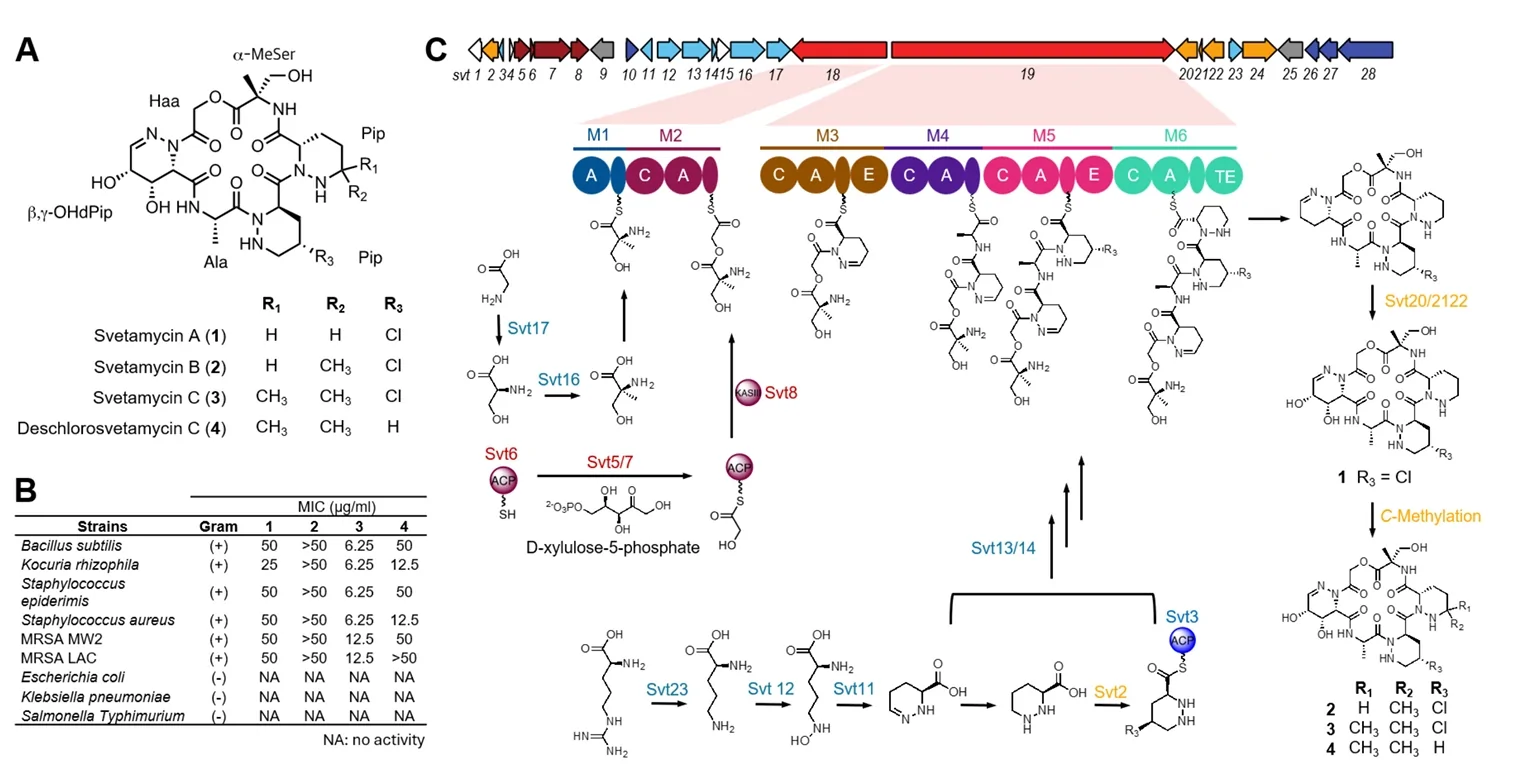
Identification of antibacterial metabolites and their associated BGC from *Streptomyces* sp. KU57. (A) Chemical structures of antibacterial metabolites svetamycins including one new (4) and three known (1–3) congeners. (B) Antibacterial assay of svetamycin congeners using 10 different pathogens. The svetamycin congeners were active against Gram-positive pathogens. Of the four congeners tested, svetamycin C (3) and deschlorosvetamycin C (4) displayed potent antibacterial activity against Gram-positive pathogens with MIC values less than 50 μg/ml. (C) The identification of svetamycin BGC and proposed biosynthesis. The svetamycin BGC was identified through whole-genome sequencing of the KU57 strain. The biosynthetic roles of individual genes were assigned based on homologous searches against the MIBiG database.

HPLC analysis of the KU57 extract revealed two major peaks clearly associated with antibacterial activity by activity-guided fractionation. Thus, we selected this strain as a case study for the isolation of antibacterial compounds. Activity-guided fractionation using a Sep-Pak C18 cartridge yielded six fractions: 0%, 20%, 40%, 60%, 80%, and 100% ACN fractions. Of these, the 40% ACN fractions exhibited the strongest antibacterial activity against *B. subtilis* (Fig. S1). HPLC analysis of this fraction identified four peaks-two major and two minor-with molecular weights of 630.2, 624.3, 644.2, and 658.2, as determined by LC-MS. These peaks were subsequently purified using semi-preparative HPLC, and their structures were elucidated by MS, and 1D and 2D NMR, including ^1^H-NMR, ^13^C-NMR, COSY, HSQC, and HMBC (Figs. S3–S17). Interpretation of these spectra identified the isolated compounds as piperazic acid-bearing natural products, svetamycins, including three previously known congeners, svetamycins A–C (1–3) (Fig. 6A) (Dardić et al., 2017). The molecular weight of 4 did not match any known svetamycin congeners, suggesting it to be a new congener. Unlike compounds 1–3, the mass spectrum of 4 lacked the [M+2]^+^ isotope peak, indicating the absence of chlorine in its structure. The ^1^H-NMR spectrum of 4 displayed two singlet methyl signals corresponding to the *δ*, *δ*-dimethyl piperazic acid found in svetamycin C (3). A comparison of the ^1^H NMR spectra of 3 and 4 revealed the absence of chlorine at the R_3_ position in 4 (Fig. S18), thus designated as deschlorosvetamycin C.

To evaluate the antibacterial activity of the isolated svetamycin congeners, we tested them against nine different bacterial pathogens, of which six are Gram-positive and three Gram-negative (Fig. 6B). Svetamycins exhibited potent antibacterial activity against Gram-positive bacteria but showed no activity against Gram-negative pathogens. Of the tested compounds, svetamycin C (3) showed the most potent antibacterial activity with a MIC value of 6.2 μg/ml against drug-sensitive pathogens and 12 μg/ml against methicillin-resistant *S. aureus* (MRSA). Deschlorosvetamycin C exhibited weaker activity than svetamycin C, suggesting the presence of chlorine contributes to the antibacterial potency.

### Identification of svetamycin BGC

Since the isolated svetamycins exhibited potent antibacterial activity against Gram-positive pathogens, identifying their BGC would offer an opportunity for structural optimization through BGC engineering (Calcott et al., 2020). Therefore, we sequenced the genome of the Svetamycin producer *Streptomyces* sp. KU57. The high-quality genome was obtained through a hybrid assembly of PacBio and Illumina sequencing reads and subsequently analyzed using antiSMASH. Given that svetamycins are peptide-based molecules with highly modified amino acids, they are likely synthesized via a non-ribosomal peptide biosynthetic system. The antiSMASH analysis identified a total of 35 BGCs, including 11 non-ribosomal peptide synthetase (NRPS) BGCs (Fig. S2). The module structure analysis of these NRPS clusters suggested that BGC region 24, designated as the *svt* cluster, is responsible for svetamycin biosynthesis (Fig. 6C and Table S2). This cluster contains two NRPS genes (*svt18/19*) together harboring six NRPS modules (M1-M2 in *svt18* and M3-M6 in *svt19*) with substrate predictions matching the structural composition of svetamycins. Additionally, genes essential for the biosynthesis of non-proteinogenic amino acids were identified within the svt cluster, including those for piperazic acid (*svt11/12/23*), α-methyl serine (*svt16/17*), and hydroxyl acetic acid (*svt5/6/7/8*). Svetamycin biosynthesis also involves hydroxylation and methylation of piperazic acid residues that are likely to occur as tailoring modifications. The *svt* cluster contains two genes predicted to encode cytochrome P450 hydroxylases, which may be responsible for hydroxylation at the first piperazic acid residue. However, no gene encoding a methyltransferase was identified in the cluster. Since there are several genes with unknown functions, it is possible that some of them would encode the necessary methyltransferase. Alternatively, the methylation of the third piperazic acid residue may be catalyzed by a methyltransferase encoded outside the *svt* cluster. Currently, we are in the process of cloning the *svt* cluster to confirm if it contains all the genes required for svetamycin biosynthesis. Cloning this cluster will also enable the diversification of svetamycin structures, facilitating studies on structure-activity relationships and target identification.

## Conclusion

The collection and preservation of indigenous biological resources have become increasingly important for nations to harness their full potential for national benefits. This is supported by the Nagoya Protocol, which came into effect on October 12, 2014, and establishes regulations for the collection and utilization of a country’s biological resources (Knauf et al., 2019). In South Korea, the National Institute of Biological Resources (NIBR) under the Ministry of Environment is responsible for maintaining the country’s native biological resources, including its actinobacterial collection. Since actinobacteria have long been an invaluable source of antibiotics, we initiated a project to explore the NIBR’s actinobacterial collection for the discovery of next-generation antibiotics. This collection encompasses genetically diverse actinobacterial species, with a predominant population of *Streptomyces* along with a small population of rare actinobacterial genera such as *Kitasatospora*, *Micromonospora*, and *Nocardioides*. Genotype-based clustering of BGCs identified from sequenced NIBR actinobacterial genomes revealed that GCFs found in multiple genomes were mostly associated with known BGCs. In contrast, unique GCFs found only in single genomes were largely cryptic, suggesting their potential as a source of novel natural products.

Our initial antibacterial activity screening of culture extracts demonstrated a high hit rate, with more than half of the strains exhibiting activity. Dereplication by LC-MS analysis indicated the presence of potentially new metabolites in many active extracts. We performed chemical investigation and whole-genome sequencing of the active strain *Streptomyces* sp. KU57, leading to the identification of svetamycins and their associated BGC. This discovery lays the foundation for future efforts in structure optimization and target identification. Overall, we have successfully established an antibacterial natural product discovery pipeline from Korean actinobacteria, integrating genomic and metabolomic analyses to facilitate the simultaneous identification of active metabolites and their associated BGCs.

## Supplementary Information

Table S1. List of strains included in the study and their closest species based on the BLAST result of 16S rRNA

Table S2. Gene annotation table for the *svt* cluster

Fig. S1. Fractionation of the KU57 extract and antibacterial activity test for the resulting fractions.

Fig. S2. antiSMASH analysis of *Streptomyces* sp. KU57 genome.

Fig. S3. LC-MS analysis of 1.

Fig. S4. ^1^H NMR spectrum (600 MHz) of 1.

Fig. S5. ^13^C NMR spectrum (150 MHz) of 1.

Fig. S6. COSY NMR spectrum (600 MHz) of 1.

Fig. S7. HSQC NMR spectrum (600 MHz) of 1.

Fig. S8. HMBC NMR spectrum (600 MHz) of 1.

Fig. S9. LC-MS analysis of 2.

Fig. S10. ^1^H NMR spectrum (600 MHz) of 2.

Fig. S11. LC-MS analysis of 3.

Fig. S12. ^1^H NMR spectrum (600 MHz) of 3.

Fig. S13. ^13^C NMR spectrum (150 MHz) of 3.

Fig. S14. LC-MS analysis of 4.

Fig. S15. ^1^H NMR spectrum (600 MHz) of 4.

Fig. S16. COSY spectrum (600 MHz) of 4.

Fig. S17. HMBC spectrum (600 MHz) of 4.

Fig. S18. A comparison of ^1^H NMR spectra between 3 and 4.
