# Supplementary material for: Exploring the biosynthetic potential of Korean Actinobacteria for antibacterial metabolite discovery
Source: J Microbiol. 2025 Sep 30;63(9):e2504002. doi: 10.71150/jm.2504002 (PMC13577020; doi:10.71150/jm.2504002)

Fig. S1. Fractionation of the KU57 extract and antibacterial activity test for the resulting fractions.

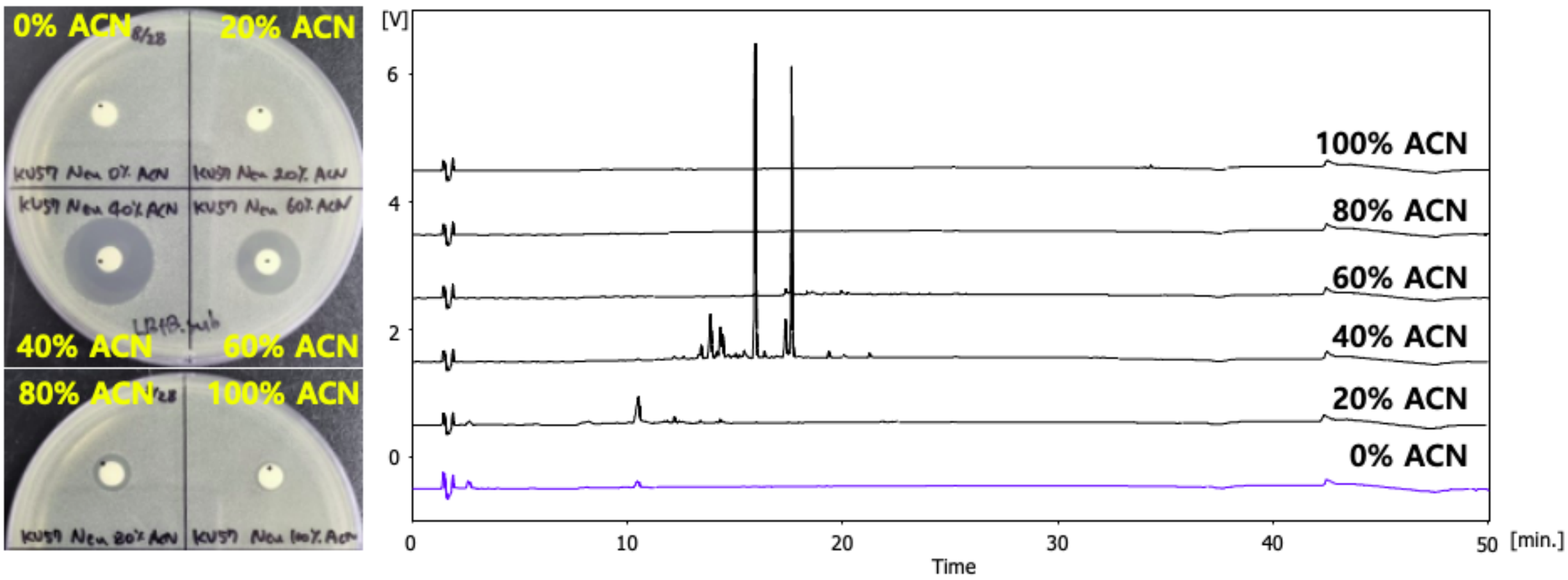

Supplement: Supplementary file 1 [file jm-2504002-Supplementary-Fig-S1.pdf]
