# Supplementary material for: Exploring the biosynthetic potential of Korean Actinobacteria for antibacterial metabolite discovery
Source: J Microbiol. 2025 Sep 30;63(9):e2504002. doi: 10.71150/jm.2504002 (PMC13577020; doi:10.71150/jm.2504002)
Supplement: Supplementary file 11 [file jm-2504002-Supplementary-Fig-S2.pdf]

Fig. S2. antiSMASH analysis of *Streptomyces* sp. KU57 genome.

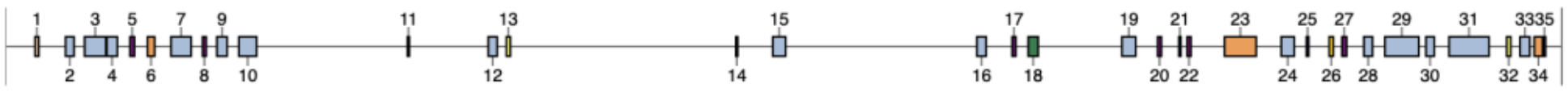

Region	Type	From	To
Region 1	indole	170,827	192,059
Region 2	T1PKS , NRPS-like	346,027	394,528
Region 3	T1PKS , other , terpene , RiPP-like , indole , NRPS	455,226	578,261
Region 4	NRPS , T1PKS	583,085	643,601
Region 5	terpene	716,420	741,913
Region 6	T3PKS	817,068	858,108
Region 7	T1PKS , NRPS-like	952,307	1,067,815
Region 8	terpene	1,133,692	1,154,261
Region 9	T3PKS , NRPS	1,215,782	1,274,727
Region 10	HR-T2PKS , NRPS , T3PKS	1,342,245	1,444,359
Region 11	ectoine	2,311,580	2,321,984
Region 12	NRPS , T1PKS	2,776,151	2,826,772
Region 13	lanthipeptide-class-iii	2,880,076	2,902,904
Region 14	melanin	4,199,318	4,209,719
Region 15	NRPS , arylpolyene	4,411,323	4,485,082
Region 16	other , T1PKS , butyrolactone	5,584,638	5,637,695
Region 17	terpene	5,786,987	5,807,937
Region 18	NRPS	5,879,873	5,936,841
Region 19	NI-siderophore , NRP-metallophore , NRPS	6,418,294	6,497,388
Region 20	terpene	6,623,630	6,644,640
Region 21	RiPP-like	6,745,752	6,757,044
Region 22	terpene	6,792,652	6,814,820
Region 23	T1PKS	7,008,082	7,189,959
Region 24	PKS-like , other , NRPS	7,333,919	7,408,798
Region 25	hydrogen-cyanide	7,478,568	7,491,582
Region 26	LAP	7,609,821	7,633,518
Region 27	terpene	7,682,794	7,709,414
Region 28	T1PKS , butyrolactone	7,808,768	7,858,575
Region 29	T3PKS , NRPS , NRPS-like	7,929,666	8,124,768
Region 30	T1PKS , NRPS-like	8,165,608	8,213,400
Region 31	NRPS-like , lanthipeptide-class-v , thiopeptide , thioamitides , T1PKS , transAT-PKS , NRPS	8,298,355	8,528,170
Region 32	lanthipeptide-class-ii	8,629,889	8,653,158
Region 33	NRPS , nucleoside	8,706,457	8,762,489
Region 34	T1PKS	8,788,779	8,836,284
Region 35	RiPP-like	8,840,947	8,852,854
