# Supplementary material for: Exploring the biosynthetic potential of Korean Actinobacteria for antibacterial metabolite discovery
Source: J Microbiol. 2025 Sep 30;63(9):e2504002. doi: 10.71150/jm.2504002 (PMC13577020; doi:10.71150/jm.2504002)

Fig. S6. COSY NMR spectrum (600 MHz) of **1**.

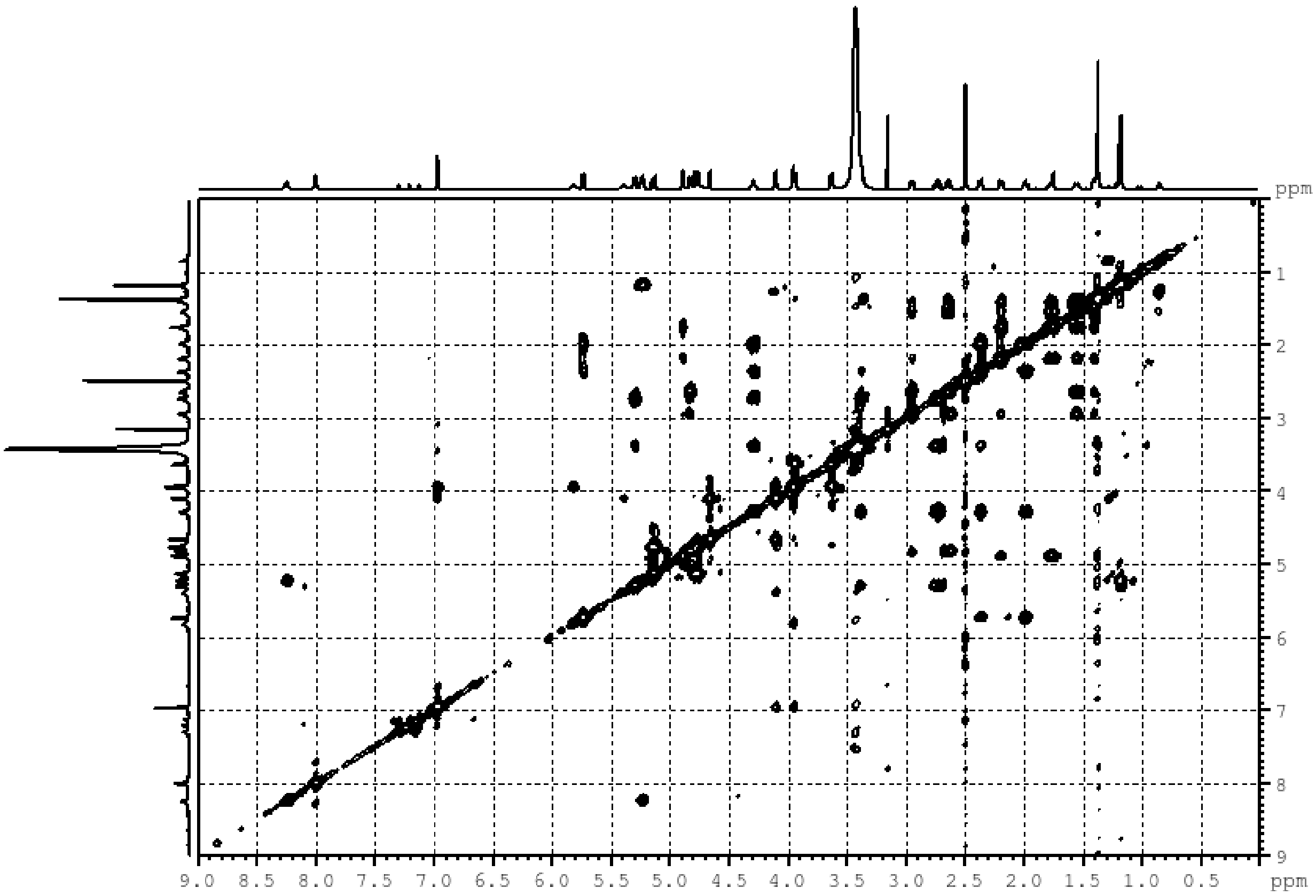

Supplement: Supplementary file 15 [file jm-2504002-Supplementary-Fig-S6.pdf]
