# Supplementary material for: Exploring the biosynthetic potential of Korean Actinobacteria for antibacterial metabolite discovery
Source: J Microbiol. 2025 Sep 30;63(9):e2504002. doi: 10.71150/jm.2504002 (PMC13577020; doi:10.71150/jm.2504002)
Supplement: Supplementary file 19 [file jm-2504002-Supplementary-Table-S1.pdf]

Table S1. List of strains included in the study and their closest species based on the BLAST result of 16S rRNA

Label	NIBR number	Closest species (identity)
KU11	NIBRBAC000498102	*Streptomyces fodineus* (99.86%)
KU12	NIBRBAC000503366	*Streptomyces guryensis* (99.93%)
KU13	NIBRBAC000506136	*Arthrobacter hankyongi* (100%)
KU15	NIBRBAC000498115	*Nocardioides anomalus* (99.93%)
KU16	NIBRBAC000506160	*Nocardioides okcheonensis* (100%)
KU17	NIBRBAC000506158	*Micromonospora antibiotica* (99.93%)
KU18	NIBRBAC000506159	
KU19	NIBRBAC000498624	*Kitasatospora acidiphila* (99.93%)
KU20	NIBRBAC000003010	*Streptomyces lydicus* (99.67%)
KU21	NIBRBAC000003011	*Paraburkholderia monticola* (99.79%)
KU22	NIBRBAC000003041	*Streptomyces shenzhenensis* (99.66%)
KU24	NIBRBAC000498263	*Streptomyces ryensis* (99.72%)
KU26		*Streptomyces mirabilis* (100%)
KU27		*Streptomyces mirabilis* (100%)
KU28		*Streptomyces mirabilis* (99.93%)
KU29		*Streptomyces atriruber* (98.75%)
KU30		*Streptomyces populi* (98.75%)
KU31		*Streptomyces lanatus* (98.82%)
KU32		*Streptomyces graminisoli* (99.24%)
KU33		*Streptomyces spiralis* (99.37%)
KU34		*Streptomyces sanqlieri* (99.37%)
KU35		*Streptomyces cyslabdanicus* (99.29%)
KU36		*Streptomyces griseoruber* (99.02%)
KU37		*Streptomyces roietensis* (99.08%)
KU38		*Kitasatospora purpeofusca* (98.91%)
KU39		*Kitasatospora purpeofusca* (98.96%)
KU41		*Kitasatospora xanthocidica* (99.23%)
KU42		*Kitasatospora cheerisanensis* (98.88%)
KU43		*Kitasatospora cheerisanensis* (99.3%)
KU44		*Kitasatospora misakiensis* (99.37%)
KU45		*Kitasatospora aureofaciens* (99.72%)
KU46		*Kitasatospora cinereorecta* (100%)
KU47		*Kitasatospora nipponensis* (100%)
KU48		*Streptomyces broussonetiae* (99.1%)
KU49		*Streptomyces rhizophilus* (99.1%)
KU50		*Streptomyces fuscichromogenes* (99.15%)
KU51		*Streptomyces alboniger* (99.17%)
KU52		*Streptomyces hyaluromycini* (99.24%)
KU53		*Streptomyces melanogenes* (99.24%)
KU54		*Nocardia tenqchongensis* (99.72%)
KU55		*Micromonospora schwarzwaldensis* (99.93%)
KU56	NIBRBAC000002097	*Streptomyces omiyaensis* (99.86%)
KU57	NIBRBAC000002345	*Streptomyces kanamyceticus* (99.79%)

Table S1. (continued)

Label	NIBR number	Closest species (identity)
KU58	NIBRBAC000002346	*Streptomyces globisporus* (99.86%)
KU59	NIBRBAC000002647	
KU60	NIBRBAC000002649	
KU61	NIBRBAC000502553	
KU62	NIBRBAC000503235	*Streptomyces globosus* (99.79%)
KU63	NIBRBAC000503273	*Streptomyces zaomyceticus* (99.86%)
KU64	NIBRBAC000503274	
KU65	NIBRBAC000503275	*Streptomyces pratensis* (99.86%)
KU66	NIBRBAC000503276	*Streptomyces amakusaensis* (99.72%)
KU67	NIBRBAC000503277	*Streptomyces anulatus* (99.86%)
KU68	NIBRBAC000503278	*Streptomyces netropsis* (99.66%)
KU69	NIBRBAC000504784	
KU70	NIBRBAC000505105	*Streptomyces mirabilis* (100%)
KU71	NIBRBAC000506140	*Streptomyces scabiei* (99.79%)
KU73	NIBRBAC000506144	
KU74	NIBRBAC000506146	*Streptomyces californicus* (100%)
KU83	NIBRBAC000508932	*Streptomyces galbus* (99.93%)
