# Supplementary material for: Exploring the biosynthetic potential of Korean Actinobacteria for antibacterial metabolite discovery
Source: J Microbiol. 2025 Sep 30;63(9):e2504002. doi: 10.71150/jm.2504002 (PMC13577020; doi:10.71150/jm.2504002)
Supplement: Supplementary file 20 [file jm-2504002-Supplementary-Table-S2.pdf]

Table S2. Gene annotation table for the *svt* cluster

Gene	Length (bp)	Proposed function	Homologous protein	Origin	Identity (aa %)	MIBiG Protein ID
Svt1	747	Thioesterase	VirJ	*Streptomyces virginiae*	28	BAF50718.1
Svt2	957	Chlorinating enzyme	NunB2	*Pseudomonas sp. In5*	61	KPN90373.1
Svt3	264	Acyl Carrier Protein (ACP)	Ecm13	*Streptomyces lasaliensis*	46	BAE98162.1
Svt4	219	MbtH-like protein	Tlo24	*Streptomyces canus*	74	ALV86870.1
Svt5	927	Dehydrogenase alpha subunit	Qcn7	*Streptomyces melanovinaceus*	71	BAO84856.1
Svt6	231	Acyl Carrier Protein (ACP)	-	*Streptomyces lusitanus*	58	AGD80610.1
Svt7	2,142	Dehydrogenase beta subunit	Qcn9	*Streptomyces melanovinaceus*	58	BAO84858.1
Svt8	1,011	3-oxoacyl-synthase III	Qcn10	*Streptomyces melanovinaceus*	68	BAO84859.1
Svt9	1,311	Hypothetical protein	NybQ	*Streptomyces albus subsp. chlorinus*	43	AYV61418.1
Svt10	696	Regulator	HmtD	*Streptomyces himastatinicus ATCC 53653*	62	CBZ42138.1
Svt11	648	Negative regulator	HmtC	*Streptomyces himastatinicus ATCC 53653*	66	CBZ42137.1
Svt12	1,350	Monooxygenase	HmtM	*Streptomyces himastatinicus ATCC 53653*	62	CBZ42147.1
Svt13	1,650	AMP-binding protein	MfnK	*Streptomyces drozdowiczii*	63	AJV88383.1
Svt14	279	Acyl Carrier Protein (ACP)	MfnL	*Streptomyces drozdowiczii*	51	AJV88384.1
Svt15	768	Type II thioesterase	RmpR	*Amycolatopsis sp.*	37	AWH12637.1
Svt16	1,977	C-methyltransferase	CysS	*Cystobacter sp. Cbv34*	40	AKP45384.1
Svt17	1,371	Hydroxymethyltransferase	AmiS	*Streptomyces vinaceusdrappus*	59	AEF16058.1
Svt18	5,514	NRPS	HmtL	*Streptomyces himastatinicus ATCC 53653*	36	CBZ42146.1
Svt19	16,353	NRPS	HmtL	*Streptomyces himastatinicus ATCC 53653*	52	CBZ42146.1
Svt20	1,224	Cytochrome P450	SceD	*Streptomyces sp. SD85*	49	ANH11399.1
Svt21	183	Ferredoxin	SceC	*Streptomyces sp. SD85*	52	ANH11398.1
Svt22	1,209	Cytochrome P450	-	*Streptomyces sp. FXJ7.023*	52	AGC09527.1
Svt23	783	Amidinotransferase	Ulm27	*Streptomyces sp. KCB13F003*	64	ATU31804.1
Svt24	1,968	Adenylation domain-containing protein	ABB07_39130	*Streptomyces incarnatus*	61	AKJ15827.1
Svt25	1,413	Beta-lactamase family protein	Mur30	*Streptomyces sp. NRRL 30471*	38	ADZ45342.1
Svt26	792	ABC transporter	STRAU_0340	*Streptomyces aurantiacus JA 4570*	43	EPH46601.1
Svt27	1,077	ABC transporter	STRAU_0339	*Streptomyces aurantiacus JA 4570*	56	EPH46600.1
Svt28	3,144	Transcriptional regulator	Sare_4552	*Salinispora arenicola CNS-205*	35	ABW00321.1
